# Effects of *Bacillus amyloliquefaciens* XJ-BV2007 on Growth of *Alternaria alternata* and Production of Tenuazonic Acid

**DOI:** 10.3390/toxins15010053

**Published:** 2023-01-07

**Authors:** Qinlan Jia, Yingying Fan, Shuaishuai Duan, Qiaomei Qin, Yu Ding, Min Yang, Yan Wang, Fengjuan Liu, Cheng Wang

**Affiliations:** 1 College of Life Science and Technology, Xinjiang University, Urumqi 830049, China; 2 Key Laboratory of Agro-products Quality and Safety of Xinjiang, Laboratory of Quality and Safety Risk Assessment for Agri-products (Urumqi), Institute of Quality Standards & Testing Technology for Agri-products, Xinjiang Academy of Agricultural Sciences, Urumqi 830091, China; 3 College of Biology and Geography Sciences, Yili Normal University, Yining 835000, China

**Keywords:** *Alternaria alternata*, *Bacillus amyloliquefaciens*, tomato, tenuazonic acid, lipopeptide, fengycin

## Abstract

Large amounts of processing tomato are grown in Xinjiang, China. Tomato black spot disease, caused by *Alternaria* spp., and the produced alternaria toxins in tomato products are posing risks to human health. In this study, we isolated a rhizospheric bacterium, XJ-BV2007, from tomato (*Solanum lycopersicum*) fields, which we identified as *Bacillus amyloliquefaciens*. We found that this bacterium has a strong antagonistic effect against *Alternaria alternata* and reduces the accumulation of alternaria toxins in tomatoes. According to the antifungal activity of the bacteria-free filtrate, we revealed that *B. amyloliquefaciens* XJ-BV2007 suppresses *A. alternata* by the production of antifungal metabolites. Combining semi-preparative high-performance liquid chromatography, we employed UPLC-QTOF-MS analysis and the Oxford cup experiment to find that fengycin plays an important role in inhibiting *A. alternata*. This paper firstly reported that *B. amyloliquefaciens* efficiently controls tomato black spot disease and mycotoxins caused by *A. alternata*. *B. amyloliquefaciens* XJ-BV2007 may provide an alternative biocontrol strain for the prevention of tomato black spot disease.

## 1. Introduction

The tomato is the world’s second most economically important vegetable crop after the potato. Because of its widespread use and high concentrations of vitamin A, C, and K; minerals; amino acids; and antioxidant lycopene, the demand for both fresh and processed tomatoes is high [1]. Fungal rots are one of the leading causes of substantial economic losses and decreases in tomato quality and nutrient content [2]. *Alternaria* spp. are opportunistic pathogens that cause a wide range of plant diseases, including black spot of tomatoes [3], olives, and carrots; black and grey rot of citrus fruits; black point of small-grain cereals; and alternaria diseases of apples [4]. Lemma et al. discovered that the average frequency of *Alternaria* incidence in infected tomato fruit samples was 37.5%; when tomato fruit becomes infected with *Alternaria alternata*, it develops dark, large, leathery, and sunken patches, which negatively impact tomato output and nutritional attributes [5]. Jiang et al. reported that *A. alternata* was the predominant species with incidence values of 65.4% (17/26) among 26 *Alternaria* strains isolated from tomatoes with black spot disease [6].

In addition to the direct economic losses caused by fungal infections, several secondary *A. alternata* metabolites are classified as mycotoxins and are as potentially harmful to human health [7]. Tenuazonic acid (TeA) has the highest detection rate and content compared with other alternaria toxins and may lead to an increased incidence of esophageal cancer in humans [8]. Alternariol (AOH) and alternariol monomethyl ether (AME) are genotoxic in bacteria and mammalian cells in vitro and have a significantly higher toxic potential [9]. The European Food Safety Authority (EFSA) highlighted the relevance of the ingestion of these compounds to human health and determined that the threshold of toxicological concern (TTC) for TeA is 1500 ng/kg b.w. per day, and 2.5 ng/kg b.w. per day for AOH and AME [10]. Fresh tomatoes and tomato-based products are major sources of dietary exposure [11]. On 5 April 2022, the European Union (EU) issued Proposal 202/553 to amend Regulation (EC) No 401/2006 on the monitoring of alternaria toxins in food, including processed tomato products, in which AOH, AME, and TeA consumption should not exceed 10, 5, and 500 μg/kg, respectively [12].

Despite widespread worry about alternaria toxicity on a global scale, tomato products contain notable amounts of the toxin. In an analysis of Argentine tomatoes and products, the alternaria mycotoxin detection rate was 28%, with TeA concentrations up to 4021 μg/kg [13]. In Brazil, the TeA detection rates in fresh tomatoes and tomato puree were 35% and 18%, respectively [14]. The detection rates of TeA, AOH, and AME in 31 samples of tomato paste purchased from a Chinese market were 100%, 45.2%, and 90.3%, respectively; the quantities were 10.20–1787.00, 2.50–300.00, and 0.32–8.00 μg/kg, respectively [15]. These findings demonstrate that the amount of alternaria toxins found is far above the current regulatory standard. All of the toxins in tomato products are derived from raw tomato materials infected with *Alternaria* spp., which were processed into the final tomato products. Thus, it is a significant task to control the *A. alternata* and alternaria toxins in raw processing tomatoes.

Direct inhibition of fungal growth and mycotoxin production on crops is widely regarded as the most effective methods to reduce the adverse effects of toxins on animals and humans [16]. Biological control, as an effective direct inhibition method, was widely used in the control of various plant pathogens in recent years because of its antimicrobial resistance ability, environmental friendliness, and lack of residual effects [17]. Among the biological control agents (BCAs) used, *Bacillus* spp. are promising. A phyllosphere bacterium, *B. subtilis* PMB102 [18], isolated from the tomato leaf, was found to inhibit the growth of *Alternaria brassicicola* ABA-31 on PDA by 56.8% and suppress the *Alternaria* leaf spot on Chinese cabbage (*Brassica rapa*). *Magnaporthe oryzae*, which causes rice blast disease, can be controlled by using *Bacillus safensis*; in field experiments, spraying with *B. safensis* B21 fermentation broth (29.48%) resulted in a significantly lower disease index compared with when carbendazim was used (37.88%) [19]. Concerning tomato black spot disease, no studies show that *Bacillus* spp. can suppress *A. alternata* growth or even reduce the mycotoxin contamination.

Xinjiang is one of the three major tomato cultivation and processing centers in the world, and its tomato products are all sold overseas. From the perspective of tomato product quality and safety, an efficient microorganism with fungistasis and detoxification abilities needs to be screened. Therefore, the aim of the present study is to isolate the antagonistic bacteria with a significant inhibitory effect on *A. alternata* and evaluate the potential of its in vitro and in vivo antifungal activity. Further research was conducted to investigate its effect on the production of mycotoxins and identify the antifungal compounds. This is critical for the control of tomato black spot disease and the alleviation of mycotoxins’ contamination, which is important for the healthy development of the processing tomato industry.

## 2. Results and Discussion

### 2.1. Antagonistic Effect of Bacterial Strains against A. alternata

**Figure 1:**
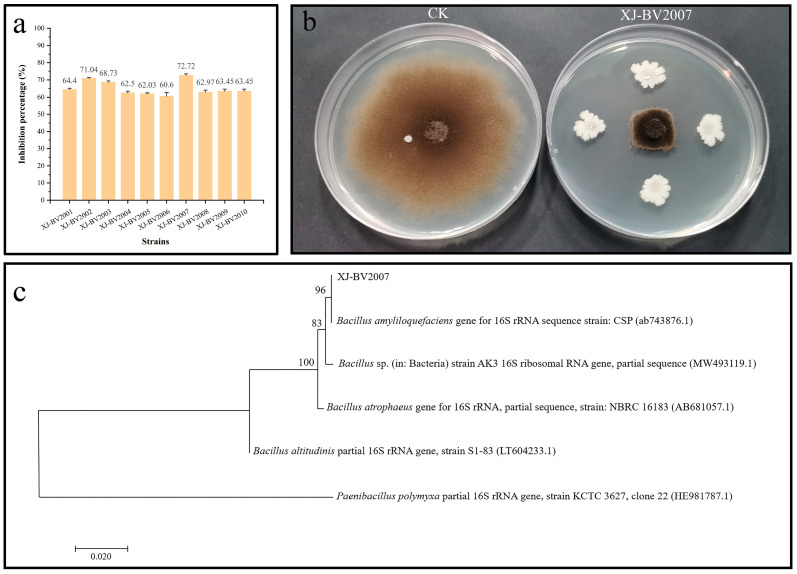
Screening and identification of antagonistic bacteria. (**a**) Antifungal activity of ten antagonistic bacteria against *A. alternata* isolate H10. (**b**) Antifungal activity of XJ-BV2007 strain against *A. alternata* isolate H10 on NA plate. (**c**) Phylogenetic tree of XJ-BV2007 based on 16S rRNA gene sequences. The numbers above the branches are confidence limits expressed as percentages.

**Figure 2:**
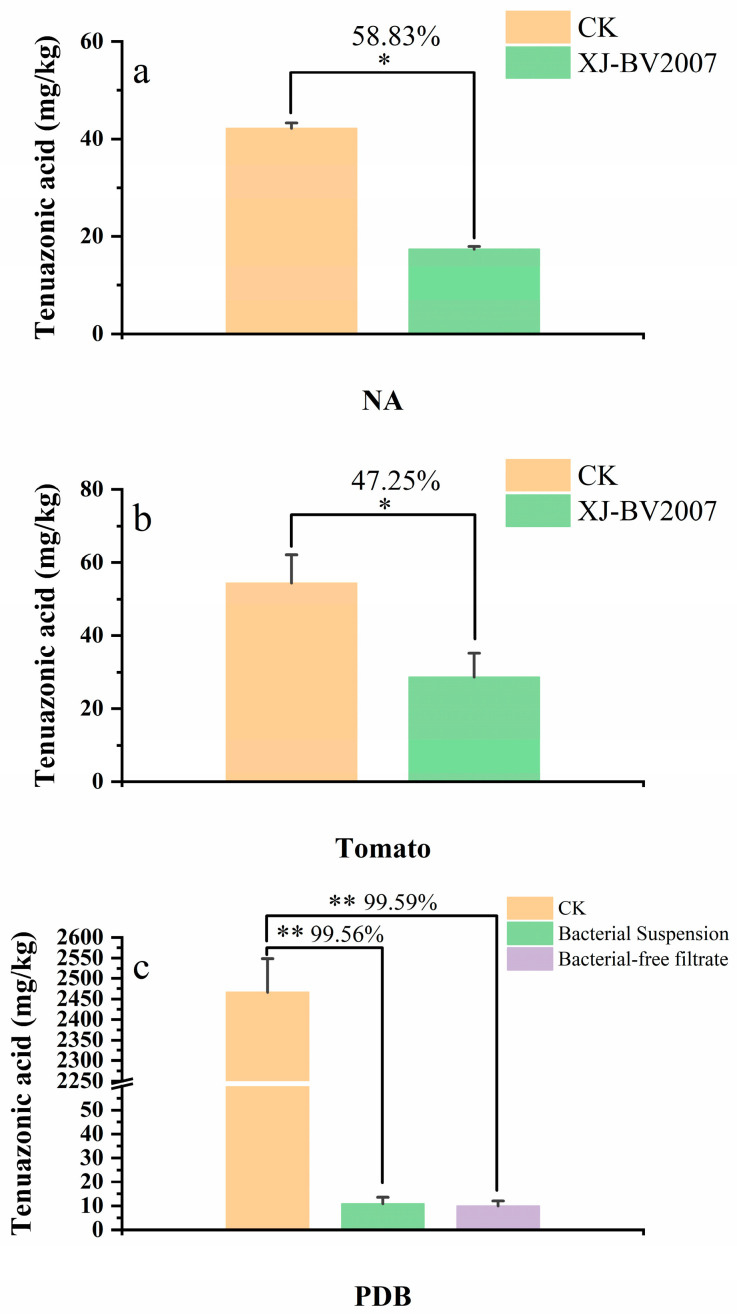
Inhibition of TeA accumulation in different experimental groups: NA medium (**a**), tomato (**b**), and PDB medium (**c**). Over the line connecting bars represents a percentage reduction in TeA concentration. Asterisks over bars represent significant differences between treatments and CK (* *p* < 0.05, ** *p* < 0.01).

Ten bacterial strains from 80 isolates were screened for potential antagonistic activity towards *A. alternata* isolate H10 in the dual culture test, as shown in Figure 1a. Among these isolates, XJ-BV2007 showed the strongest antagonistic activity against *A. alternata* isolate H10, and the percentage of growth inhibition after seven days of incubation was approximately 72.72% (shown in Figure 1a). In addition, as shown in Figure 1b, after 7 days of standoff incubation, there was little growth of *A. alternata* isolate H10 on the NA plate inoculated with XJ-BV2007 compared to the CK. Compared to other alternaria toxins, TeA has the highest detection rate and content in tomatoes and their products by our research and other literature [8]. Thus, TeA was mainly detected in this study. Moreover, upon treatment with XJ-BV2007 after 7 days, there was a 58.83% (Figure 2a) reduction in TeA production when compared to the control group (CK). Therefore, XJ-BV2007 was selected to conduct further experiments.

### 2.2. Molecular Characterization by 16S rRNA Gene Analysis

The XJ-BV2007 strain is a Gram-positive, rod-shaped bacterium and can form milk-white, opaque colonies on a NA plate. The nucleotide sequence of the 16S rRNA gene of the XJ-BV2007 strain was sequenced by Sangon Biotech and obtained a 1362-bp gene. The nucleotide sequences queried against the NCBI Gene Bank database were aligned by the online BLAST program. Using the MEGA 7.0 program to construct a neighbor-joining phylogenetic tree, the evolutionary history was inferred by the neighbor-joining algorithm (Figure 1c). The result showed that the XJ-BV2007 strain exhibited a 96% sequence identity to *B. amyloliquefaciens*. According to the 16S rRNA gene sequence analysis, this isolated strain was therefore named as *B. amyloliquefaciens* XJ-BV2007, which was to submitted to the China General Microbiological Culture Collection Center for storage, and the preservation number is CGMCC NO. 23,669. In the acute oral toxicity test, the KM mice showed no signs of toxicity or death within 5 days of exposure. At the end of the test, no abnormalities were observed in the gross pathology of the test animals. The experimental results showed that the acute oral toxicity of the cell suspension of XJ-BV2007 to KM mice was LD_50_ > 5000 mg/kg·bw, which is actually non-toxic in accordance with China’s agricultural industry standards. Chen et al. isolated *B. amyloliquefaciens* PG12 and was able to reduce the mycelial growth of *Botryosphaeria dothidea* on PDA [20]. The growth of *A. alternata* on PDA medium was also effectively inhibited by *Bacillus velezensis* [21]. These results were in agreement with this work that isolated *B. amyloliquefaciens* XJ-BV2007 and was able to prevent *A. alternata* growth on solid medium.

### 2.3. Co-Inoculation of Antagonistic Microorganisms and A. alternata in Tomato

**Figure 3:**
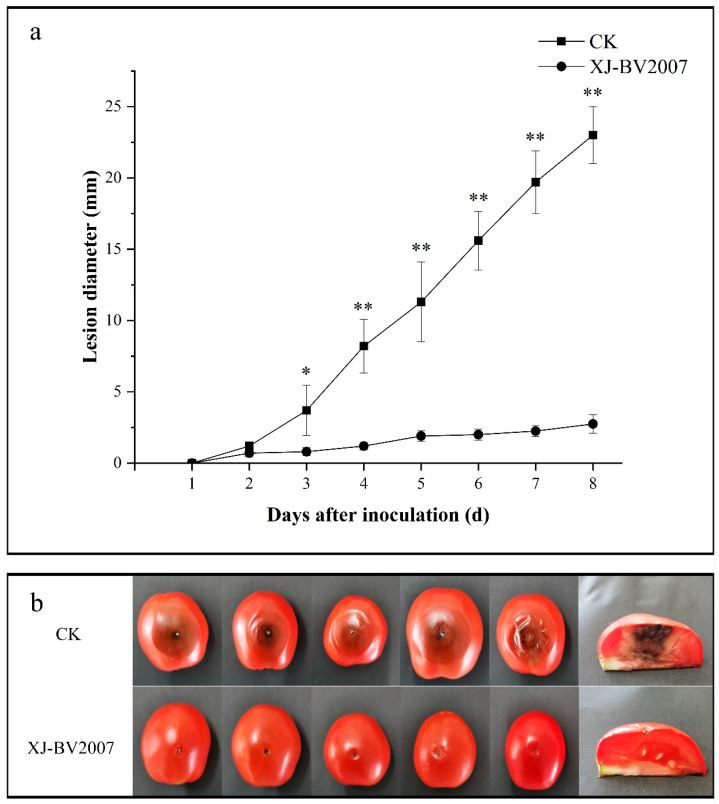
Antifungal potential of *B. amyloliquefaciens* XJ-BV2007 against *A. alternata* on tomato. (**a**) The condition of tomato black spot disease in the experimental group and CK group at 8 days. (**b**) The lesion diameter in the experimental group and CK group during 8 days of storage. Asterisks over bars represent significant differences between treatments and CK (* *p* < 0.05, ** *p* < 0.01).

Further antifungal experiments were conducted by co-inoculation of *B. amyloliquefaciens* XJ-BV2007 with *A. alternata* isolate H10 on the tomato. During 8 days of storage, the *B. amyloliquefaciens* XJ-BV2007 suspension significantly decreased the disease severity caused by *A. alternata* isolate H10, as shown in Figure 3a. After 8 days of storage, fruits inoculated with the pathogen showed a mean lesion diameter of 23 mm; however, tomatoes treated with the XJ-BV2007 bacterial suspension had a mean lesion diameter of 2.75 mm (Figure 3b), representing an 88.04% reduction in the lesion diameter. As was expected, after artificial inoculation with the *A. alternata* isolate H10, the TeA levels of tomato fruits treated with XJ-BV2007 were 47.25% lower than those of the untreated tomato after 8 days of storage (Figure 2b and Figure S1), indicating that XJ-BV2007 significantly (*p* < 0.05) inhibited the TeA accumulation.

These findings are significant since the treatments were effective in preventing *A. alternata* fungal infection on tomato fruits during postharvest, suggesting the potential of *B. amyloliquefaciens* XJ-BV2007 as a biocontrol agent against *A. alternata*. Some authors have demonstrated that many species of *B. amyloliquefaciens* are effective in the biocontrol of various fungal phytopathogens. Calvo et al. reported that *B. amyloliquefaciens* was able to reduce *Penicillium expansum* incidence in apples from 100% to 20% [22]. Nevertheless, few authors have focused on the impact of antagonistic bacteria on the accumulation of mycotoxins. In the work of Wachowska et al., antagonistic yeasts considerably reduced moniliformin (MON) and enniatin (ENNs) concentrations by 7.67–92.87% and prevented the accumulation of mycotoxins in wheat grain in the long term [23]. Up to now, research on the inhibitory effects of antagonistic bacteria on toxins in tomatoes has not been reported. Since *Alternaria alternata* grows and causes black spot disease both in the growing and post-harvest storage stages of tomatoes, XJ-BV2007 is hoped to be used in both.

### 2.4. Extraction, Purification, and Identification of Lipopeptides Produced by B. amyloliquefaciens XJ-BV2007

#### 2.4.1. Antifungal Activity by Bacteria-Free Filtrate

**Figure 4:**
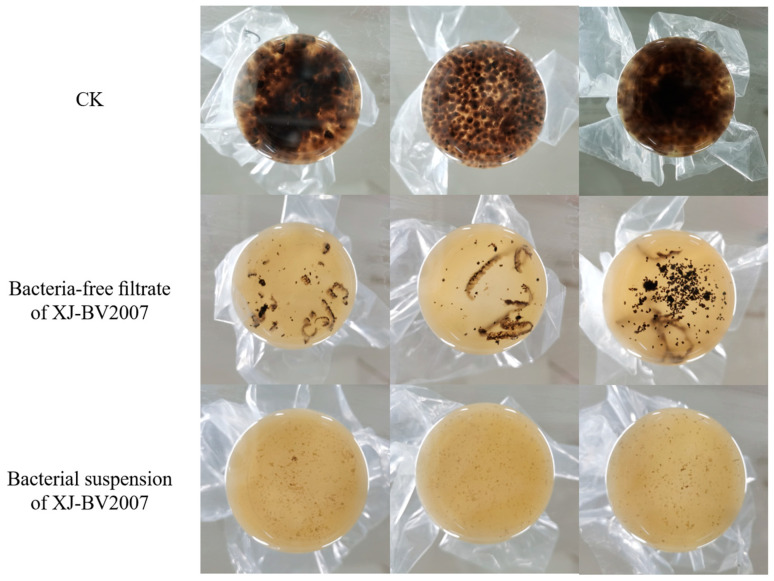
Antifungal activity of bacteria-free filtrate and bacterial suspension of *B. amyloliquefaciens* XJ-BV2007 on PDB liquid medium against *A. alternata*.

To determine further whether the antifungal activity of isolate XJ-BV2007 depends on the cell viability or not, comparative experiments were conducted with viable cells and the cell-free culture supernatant. As shown in Figure 4, both the bacterial suspension and the cell-free filtrate have a substantial inhibitory effect on the growth of *A. alternata* isolate H10 in PDB liquid medium. It is noteworthy that there was a 99% reduction in TeA accumulation by both the bacterial suspension and the cell-free filtrate when compared to untreated, shown in Figure 2c.

*Bacillus* spp. are well-known producers of biologically active compounds with potent antifungal activities. According to Calvo et al., cells, endospores, and cell-free supernatants of *B. amyloliquefaciens* BUZ-14 show strong in vitro antifungal activity against various fungi, and they consider that this ability is due to their wide range of antifungal compounds [22]. Other studies reported the crude lipopeptides extracted from the *B. amyloliquefaciens* TF28 culture filtrate against a variety of fungi, such as *Fusarium moniliforme*, *Botrytis cinerea*, and *Fusarium oxysporum* [24]. The in vitro antifungal activity of XJ-BV2007 cell-free supernatants was equal to or better than that of cells. Therefore, we can propose that metabolites produced by XJ-BV2007 inhibited spore germination of *A. alternata*.

#### 2.4.2. PCR Amplification of Lipopeptide Biosynthetic

It is well known that lipopeptides, with efficiently antagonistic activities, are metabolites produced by *Bacillus* spp. Based on their structure, lipopeptides are grouped into three families: iturins, fengycins, and surfactins [25]. In order to determine which type of lipopeptide the active substance in XJ-BV2007 is, the *ItuA*, *ItuD*, *SrfAC*, *SrfAB*, *FenB*, and *FenC* primers were utilized for detection of the lipopeptide synthetase genes (*ItuA*, *ItuD*, *srf*, and *fen*) in our study. The results shown in Table 1 and Figure S2 indicated that *B. amyloliquefaciens* XJ-BV2007 has the genes for producing iturin A, surfactin, and fengycin.

It was reported that iturins and fengycins exhibited strong antifungal activities, but surfactins exhibited an antibacterial effect and a low fungitoxicity [26,27]. Previous reports showed that *B. atrophaeus* CAB-1 suppressed the plant pathogen Botrytis cinerea by producing fengycin [28], and *B. atrophaeus* OSY-7LA could be used in food preservation by producing subtilosin, surfactin and plipastatin [29].

#### 2.4.3. Antifungal Activity by Crude Lipopeptides

**Figure 5:**
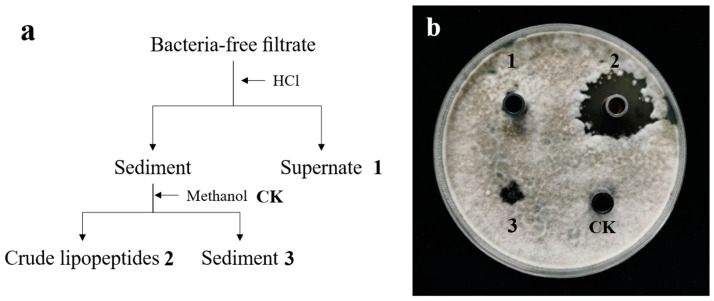
Extraction of antifungal compounds (**a**) and Oxford Cup experiment of different extracts (**b**).

The PCR results showed the presence of three lipopeptide gene clusters for surfactin, fengycin, and iturin at the genomic level, so the crude lipopeptide was extracted from the bacteria-free filtrate broth according to Kim et al. [30], and their antifungal activity against *A. alternata* isolate H10 was evaluated by the Oxford Cup Experiment. As shown in Figure 5b, we can see that crude extract 2 has a significant inhibitory effect on *A. alternata* isolate H10; extract 1 and methanol CK were barely effective, but extract 3 has a little inhibitory effect, which may be caused by insufficient methanol extraction.

#### 2.4.4. Purification of Lipopeptides

In order to determine the antifungal compounds, the above methanol extract of *B. amyloliquefaciens* XJ-BV2007 was separated and purified using semi-preparative high performance liquid chromatography. Since there is no suitable standard for lipopeptides, the fractions were collected in a time sequence rather than by the retention time of the substances. Thirty fractions, collected every one minute, were condensed and assayed for their antifungal activity against *A. alternata* isolate H10. Fifteen fractions with statistically significant biological activity were collected between retention times of 14 to 20 min and 22 to 29 min. The fractions between the elution time of 1 to 13 min, 21 min, and 30 min were not significantly active against *A. alternata* isolate H10 (Table 2 and Figures S3–S17).

#### 2.4.5. Identification of Lipopeptides Produced by *B. amyloliquefaciens* XJ-BV2007

To screen for the target antifungal compounds, an in-house library was created by uploading the mol files of the 34 lipopeptides obtained from the ChemSpider database (http://www.chemspider.com, accessed on 4 July 2022) to the UNIFI software, which provided their expected molecular mass and fragment ion *m*/*z* values. Subsequently, once the extracted sample solution was injected, the detailed molecular mass (to at least four decimal places), retention time, and fragment ion spectra were observed through HDMSE data analysis. The UNIFI software could automatically identify the target 34 lipopeptides by comparing the observed data to the expected data in the library.

Table S1 illustrates the identified and quantified different lipopeptides’ components using UPLC-QTOF/MS, including surfactin A (*m*/*z* 1008.6589 (M + H)^+^, iturin A2 (*m*/*z* 1043.5508 (M + H)^+^, 1081.5161 (M + K)^+^), iturin A4 (*m*/*z* 1057.5687 (M + H)^+^, 1095.5139 (M + K)^+^), iturin A6 (*m*/*z* 1071.5829 (M + H)^+^), and fengycin (*m*/*z* 1463.8024 (M + H)^+^, 1480.8289 (M + NH_4_)^+^). Fengycin was always detected in the fractions with an obvious fungistasis effect. These findings suggest that XJ-BV2007 produced all the three lipopeptides’ families, but fengycin is the main lipopeptide responsible for antifungal activity against *A. alternata* isolate H10. Several investigations have shown that LC-QTOF-MS could be used to identify lipopeptides. Ravi et al. identified surfactin C16 derivatives with *m*/*z* 1050 [M + H]^+^ from endophytic *Bacillus* sp. Fcl1 [31]. Colistin is a cyclic lipopeptide produced by the *Bacillus polymyxavar*, and Gikas et al. developed and validated a new UPLC–ESI HRMS/MS method for the determination of colistin in plasma samples from patients [32].

Fengycin is composed of a hydroxy fatty acid chain containing 16–18 carbon atoms and a polypeptide of 10 amino acid residues [33], synthesized by nonribosomal peptide synthetases (NRPSs) [34], which is the dominant lipopeptide in *Bacillus* spp. and active against a range of phytopathogenic fungi. *B. velezensis* SL-6 [21], which was isolated from fresh water samples, exhibits potent antifungal activity against *Botrytis cinerea*, and its antifungal compound was identified as fengycin. *B. subtilis* NCD-2 could produce the lipopeptides surfactin, fengycin, and iturin A, but only the fengycin had strong antifungal activity and a significant role in the biocontrol potential against cotton seedling damping-off disease, which was definitively supported by disruption of the fengycin synthetase gene [35].

## 3. Conclusions

In this study, we isolated and identified *B. amyloliquefaciens* XJ-BV2007, which could effectively inhibit the radial growth of *A. alternata* and reduce the accumulation of TeA on PDA plates and tomatoes. The results of an integrated approach combining semi-preparative high-performance liquid chromatography and the Oxford cup experiment indicated that fifteen fractions showed a significant fungistatic effect. UPLC-QTOF-MS analysis identified that the antifungal compounds against *A. alternata* produced by *B. amyloliquefaciens* XJ-BV2007 were fengycin. In summary, *B. amyloliquefaciens* XJ-BV2007 shows considerable potential as a biocontrol agent to control tomato black spot disease.

## 4. Materials and Methods

### 4.1. Chemicals and Reagents

LC-MS grade acetonitrile, formic acid, and acetic acid were purchased from Thermo Fisher Scientific (Waltham, MA, USA). Ultrapure water was provided by Watsons (A.S. Watson Group Ltd., Hong Kong, China). Standard tenuazonic acid (TeA) was purchased from Romer Labs (Romer Labs Division Holding GmbH, Getzersdorf, Austria). Leucine enkephalin was acquired from the Waters Corporation (Milford, MA, USA). The Bacteria DNA Kit was provided by TIANGEN (TIANGEN Biotech CO., LTD, Beijing, China). Potato Dextrose Agar (PDA) was purchased from Land Bridge (Beijing Land Bridge Technology Co., Ltd., Beijing). Nutrient Agar (NA) or Nutrient Broth (NB) was purchased from Hobepio (Qingdao Hope Bio-Technology Co., Ltd., Qingdao, China).

### 4.2. Instruments

The samples were homogenized with A11 basic ultra-turrax (IKA, Staufen, Germany) and weighed on an XSE204 balance (Mettler-Toledo, Greinfesee, Switzerland), mixed using an MS3 vortex mixer (IKA, Staufen, Germany), and shaken in an automatic horizontal shaker (Hannuo Instruments, Shanghai, China). We performed centrifugation with a Sorvall Biofuge Stratos system (Thermo Fisher Scientific, Waltham, MA, USA). The PCR reaction was performed using an Agilent Sure Cycler 8800 (Agilent, Santa Clara, CA, USA) and results were visualized using Bioshine GelX1520 (Bioshine, Shanghai, China)

TeA was quantified with a Waters Acquity UPLC-tandem quadrupole (TQD) mass spectrometer (Waters Corporation, Milford, MA, USA), which contained an Acquity UPLC HSS T3 (1.8 µm, 2.1 mm × 100 mm) column for separation.

Purification of crude lipopeptides was used with the UltiMate 3000 (Thermo Fisher Scientific, Waltham, MA, USA), which contained a Hypersil PREP HS C18 HPLC (5 µm, 10 mm × 250 mm) column for separation. In addition, the 34 lipopeptides were screened using a Waters Acquity UPLC ion mobility quadrupole time-of-flight mass spectrometer (Waters Corporation, Milford, MA, USA), which contained an Acquity UPLC HSS T3 (1.8 µm, 2.1 mm × 100 mm) column for separation.

### 4.3. Materials

*A. alternata* isolate H10 was formerly isolated from the tomato fruit [36]. Unless otherwise indicated, the strains were cultured on potato dextrose agar (PDA) at 27 °C.

Soil samples were collected from tomato fields at Yanqi Basin, Xinjiang in August–September at a temperature of 28–31 °C and obtained from the layer, approximately 3 cm apart on the topsoil [37]. Tomatoes for the experiment were harvested in the Changji Hui Autonomous Prefecture, where they were free of visible wounds and uniform in size and maturity.

Ten strains of antagonistic bacteria isolated from the above soil were stored at −20 °C in sterilized glycerol (30% (*v*/*v*)) and activated with nutrient agar (NA) or nutrient broth (NB) medium at 27 °C for 48 h before use. *B. amyloliquefaciens* XJ-BV2007 used in 2.6.1 was cultured on a synthetic medium containing peptone (11.25 g), yeast powder (3.75 g), starch soluble (5 g), and NaCl (1 g) per liter, at 27 °C for 48 h in a rotatory shaker with constant shaking at 130 rpm/min [19].

### 4.4. Isolation, Screening, and Identification of B. amyloliquefaciens XJ-BV2007

#### 4.4.1. Isolation of Strains

A total of 5 g of soil samples were suspended in 45 mL sterile distilled water by shaking at 27 °C 150 r/min for 30 min, and then diluted from 10^−1^ to 10^−6^ with sterile distilled water. After that, 100 μL of each dilution were incubated for 48 h on NA medium at 27 °C. Single colonies on the plate were picked, followed by streak-inoculating on NA medium.

#### 4.4.2. Dual Culture Bioassays

To screen for antagonistic bacteria against *A. alternata*, mycelial agar plugs with 6 mm diameter cutting from the culture edge containing 7-day-old *A. alternata* isolate H10 were placed at the center of the NA medium; then, the above purified bacteria were inoculated at 4 equidistance sites 1.5 cm from the center. The control samples consisted of NA medium with only *A. alternata* isolate H10. The medium was incubated for 7 days at 27 °C [38]. Fungal growth inhibition was evaluated by measuring the diameter of the fungal colony and expressed with the following equation:

Inhibition percentage (%) = [(d − d_0_)/d] × 100%

where d (mm) represents the radial growth radius of the *A. alternata* colony in the control group, and d_0_ (mm) represents the radial growth radius of the *A. alternata* colony in the experimental group.

#### 4.4.3. Detection of Alternaria Toxins

Alternaria toxins were extracted via the QuEChERS method, and quantified by ultra-performance liquid chromatography coupled to the tandem mass spectrometry (UPLC-MS/MS) [39], with minor modification. Briefly, place the above NA medium and approximately 75 mL of liquid nitrogen in a grinding mill to grind and pulverize rapidly to a 200 mesh. Mix 1.0 g of the fine homogenized sample with 10 mL of acetonitrile containing 1% acetic acid, and shake the mixture on an automatic horizontal shaker at 2500 rpm for 5 min. After centrifugation at 5000× *g* for 5 min, evaporate the supernatant to near dryness under a stream of nitrogen at 40 °C. Finally, add 1 mL of the combined solution (acetonitrile/methanol/formic acid, 70:29:1, *v*/*v*/*v*) to the residue, which was vortexed and filtered through a 0.22 μm PTFE filter.

The detection of alternaria toxins was quantified with the Waters Acquity UPLC-tandem quadrupole (TQD) mass spectrometer containing an Acquity UPLC HSS T3 column for separation. Set the column temperature to 40 °C. The mobile phase comprised acetonitrile containing 0.1% formic acid as eluent A and ultrapure water containing 0.1% formic acid as eluent B. A gradient elution was applied as follows: 5% A was initially used and linearly increased to 80% within 2.5 min, then to 90% within 2 min, then maintained for 1.5 min, after which column re-equilibration took place, leading to a total run time of 6 min. Set the flow rate to 0.3 mL/min.

Operate the MS/MS analysis in the positive mode at a capillary voltage of 3.0 kV, a desolvation temperature of 350 °C, a source block temperature of 125 °C, a desolvation gas of 800 L/h, and a cone nitrogen gas flow of 50 L/h. The collision gas was argon with a pressure of 4 × 10^−3^ mbar. We completed quantitation based on a standard curve generated by serial dilutions of 200–1000 ppb of TeA, and we calculated the percentage reduction in TeA concentration using the following equation:

The percentage reduction in TeA concentration (%) = [(C_0_ − C)/C_0_] × 100%

where C_0_ is the TeA concentration in the control group, and C is the TeA concentration in the experimental group.

#### 4.4.4. Molecular Characterization by 16S rRNA Gene Analysis

Genomic DNA of *B. amyloliquefaciens* XJ-BV2007 was isolated and purified using the TIAN amp Bacteria DNA Kit. The polymerase chain reaction (PCR) of the 16S rRNA gene was amplified using universal primers 27F (5′-AGAGTTGATVATGGCTCAG-3′) and 1492R (5′-CTACGGTTACCTT GTTACGAC-3′) [40]. PCR amplification of the 16S rRNA gene used a 50 μL reaction mixture containing 2 μL of genomic DNA of *B. amyloliquefaciens* XJ-BV2007 (0.1 μg/μL), 4 μL of each primer (100 μM), 25 μL of Taq polymerase, and 15 μL of ultrapure water. Reactions were carried out according to the following protocol: 94 °C for 2 min, 35 cycles at 94 °C for 30 s, annealing 55 °C for 20 s, with a final extension at 72 °C for 10 min. The PCR amplicons were analyzed through gel electrophoresis on a 1% (*w*/*v*) agarose gel with 1 × TBE buffer at a constant voltage of 120 V for 30 min, followed by visualization using a gel documentation system.

Amplification products were sequenced by Sangon Biotech Co. Ltd. (Shanghai, China). The sequence identity of the obtained fragment was compared to the NCBI Gene Bank database using the Nucleotide Basic Local Alignment Search Tool (BLAST) search algorithm. The 16S rRNA gene sequence-based neighbor-joining phylogenetic tree was constructed using the Molecular Evolutionary Genetics Analysis software MEGA 7.0 program following the procedure of multiple sequence alignments and comparisons Align by Clustal W [41].

#### 4.4.5. Acute Oral Toxicity Test of *B. amyloliquefaciens* XJ-BV2007

We commissioned Qingdao Sci-tech Innovation Quality Testing Co., Ltd. to conduct an acute oral toxicity test on the cell suspension of *B. amyloliquefaciens* XJ-BV2007. Twenty SPF KM mice, half male and half female, weighing 18–22 g, were used. The test was performed by gavage at a dose of 5000 mg/kg-bw in a volume of 0.4 mL/20 g-bw. The mice were observed for 3 h after administration and then once daily for 5 days. Gross pathological observations were made on mice that were near death and on mice that were executed at the end of the test [42].

### 4.5. Co-Inoculation of Antagonistic Microorganisms and A. alternata in Tomato

The postharvest biocontrol experiment was conducted according to Estiarte et al. [43], with some modifications. Tomatoes were successively rinsed three times with sterile distilled water, wiped with 75% ethanol, and inoculated with 10 μL of the *B. amyloliquefaciens* XJ-BV2007 suspension (10^5^ CFU/mL); meanwhile, 10 μL of distilled water served as a control. After 30 min, 10 μL of *A. alternata* isolate H10 (10^5^ CFU/mL) were inoculated into each wound. The tomatoes were then placed in plastic boxes for 8 days at 25 °C. Lesion diameters were measured every day. Five tomatoes were used for each replicate, and the experiments were repeated three times. Fungal growth inhibition was evaluated by measuring the diameter of the fungal colony and expressed with the following equation:

Inhibition percentage (%) = [(d − d_0_)/d] × 100%

where d_0_ (mm) represents the average lesion diameter in wounds treated with the antagonist along with *A. alternata*; d (mm) represents the average lesion diameter in the control group.

For assessment of in vivo alternaria toxins’ production, the whole artificially inoculated tomato fruits were homogenized with a blender at room temperature. Alternaria toxins were extracted via the QuEChERS method, with minor modifications [39]. Briefly, mix an amount of 5.0 g of fine homogenized samples with 5 mL of water, and then add 10 mL of acetonitrile containing 1% acetic acid. Shake the mixtures on an automatic horizontal shaker at 2500 rpm for 5 min to disperse the sample fully. Subsequently, add 4 g of anhydrous MgSO_4_ and 1 g NaCl immediately added while vigorously shaking the tube to prevent agglomeration of the salts. After centrifugation at 5000× *g* for 5 min, evaporate the supernatant to near dryness under a stream of nitrogen at 40 °C. Finally, add 1 mL of the combined solution (acetonitrile/methanol/formic acid, 70:29:1, *v*/*v*/*v*) to the residue, which is then vortexed and filtered through a 0.22 µm PTFE filter.

TeA was analyzed and quantified by UPLC-MS/MS described in Section 4.4.3.

The percentage reduction in TeA concentration was expressed as the following equation.

The percentage reduction in TeA concentration (%) = [(C_0_ − C)/C_0_] × 100%

where C_0_ is the TeA concentration in the control group, and C is the TeA concentration in the experimental group.

### 4.6. Extraction, Purification, and Identification of Antifungal Compounds Produced by XJ-BV2007

#### 4.6.1. Antifungal Activity by Bacteria-Free Filtrate

The XJ-BV2007 suspension (10^5^ CFU/mL) or XJ-BV2007 bacteria-free filtrate and spore suspension of *A. alternata* isolate H10 (10^5^ CFU/mL) were mixed into 6 mL of potato dextrose broth (PDB) liquid medium. Sterile PDB with a spore suspension of *A. alternata* isolate H10 (10^5^ CFU/mL) served as the control (the liquid composition of the control and experimental groups are shown in Table 3). The culture was subsequently incubated for 5 days on a rotary shaker incubator (120 rpm/min) at 28 °C in the dark [44].

Alternaria toxins were extracted via the QuEChERS method, with minor modification [39]. One mL of the XJ-BV2007 bacterial suspension underwent centrifugation at 10,000× *g* for 5 min, and then 200 μL of the supernatant were taken, mixed with 400 μL of acidic (about 0.5% of 1 M hydrochloric acid) ethyl acetate for extraction. After being centrifuged at 10,000× *g* for 2 min, the supernatant was evaporated to near dryness under a stream of nitrogen at 40 °C. Finally, 1 mL of the combined solution (acetonitrile/methanol/formic acid, 70:29:1, *v*/*v*/*v*) was added to the residue, which was vortexed and filtered through a 0.22 μm PTFE filter.

TeA was analyzed and quantified by UPLC-MS/MS described in Section 4.4.3.

#### 4.6.2. PCR Detection of Genes Encoding for the Lipopeptides Biosynthesis

Each PCR reaction was performed in an Agilent Sure Cycler 8800 using a 50 μL reaction mixture containing 2 μL of genomic DNA of *B. amyloliquefaciens* XJ-BV2007 (0.1 μg/μL), 4 μL of each primer (100 μM), 25 μL of Taq polymerase, and 15 μL of ultrapure water. Reactions were carried out according to the following protocol: 94 °C for 4 min, 35 cycles at 94 °C for 30 s, annealing for 30 s for each primer, and 72 °C for 45 s, with a final extension at 72 °C for 10 min [45]. The details of primers and expected amplicon sizes are provided in Table 4. The PCR amplicons were analyzed through gel electrophoresis on a 1% (*w*/*v*) agarose gel with 1 × TBE buffer at a constant voltage of 120 V for 30 min, followed by visualization using a gel documentation system.

#### 4.6.3. Extraction of antifungal compounds

The extraction method of extracellular metabolites refers to Kim et al. [30], with some modifications. The XJ-BV2007 cell-free supernatant was adjusted to pH 2.0 with concentrated HCl and kept at 4 °C overnight. Precipitated substances were collected by centrifugation (10,000× *g*, 10 min, 20 °C) and extracted with 3 volumes of methanol at least twice, then dried by a rotary evaporator at 40 °C, and ultimately, crude lipopeptides were obtained.

The antifungal activity of crude lipopeptides against *A. alternata* was tested by Oxford Cup. A total of 100 μL of the *A. alternata* isolate H10 (10^5^ CFU/mL) spore suspension were poured onto the PDA plate, spread out evenly, and left for 30 min. Oxford cups were placed and respectively filled with 100 μL of two crude extracts, coating with 100 μg of sediment and methanol as a control, shown in Figure 5a. The PDA plates were incubated at 28 °C for 5 days [18].

#### 4.6.4. Purification of Antifungal Compounds

Crude lipopeptides were purified by semi-preparative high performance liquid chromatography using a PREP HS C18 (5 μm, 10 × 250 mm) column. The mobile phase consisted of methanol and water (80:20, *v*/*v*) with a flow rate of 3 mL/min, a total run time of 30 min. Each eluate was collected every 1 min and assayed for antagonistic activity against *A. alternata* by the Oxford Cup, described in Section 4.6.3.

#### 4.6.5. Identification of Antifungal Compounds Produced by *B. amyloliquefaciens* XJ-BV2007

The identification of eluted fractions with antifungal activity was carried out on a Waters Acquity UPLC ion mobility quadrupole time-of-flight mass spectrometer (UPLC-IMS QTOF MS). For separation, a UPLC HSS T3 (1.7 μm, 2.1 × 100 mm) column was used. The mobile phase comprised acetonitrile as eluent A and ultrapure water as eluent B. Set the flow rate to 0.3 mL/min [47].

The IMS QTOF MS analysis was conducted in positive ion mode (ESI+) at a capillary voltage of 3.5 kV, a desolvation temperature of 370 °C, a source temperature of 115 °C, a desolvation gas flow of 650 L/h, and a cone nitrogen gas flow of 45 L/h. The injection volume was 10 μL. The acquisition mode was High-definition mass spectrometry (HDMS^E^), with low and high collision energies set to 6 eV and 30–60 eV, respectively. The data were acquired between 50 and 1500 *m*/*z*. Every 5 min, a leucine enkephalin solution (0.2 ng/L) was infused into the ion source to perform lock mass correction (*m*/*z* 556.2766 used as the reference ion in the positive mode). Using a Major Mix IMS/TOF Calibration Kit (Waters Corporation), ion mobility and mass calibration were carried out. The UNIFI 1.8.2 software (Waters Corporation, Milford, MA, USA) was used to perform the other data acquisition and processing settings, which were set in accordance with the manufacture’s guideline.

### 4.7. Statistical Analysis

All the experiments were performed in triplicate for each treatment, and each replicate included three determinations. Statistical analysis was performed with SPSS 18.0. Analysis of variance (ANOVA) was used to determine the effects of the treatments, and Duncan’s multiple range tests were used to compare the means. Differences at *p* < 0.05 were considered as significant. Asterisks over bars represent significant differences between treatments and CK (* *p* < 0.05, ** *p* < 0.01)

## Figures and Tables

**Table 1:** PCR detection of lipopeptides’ biosynthesis genes from *B. amyloliquefaciens* XJ-BV2007.

Antibiotic	Genes	Detection in XJ-BV2007
Bacillomycin D	*bamC*	−
	*bamD*	−
Iturin A	*ituD*	+
	*ituA*	+
	*ituC*	−
Surfactin	*srfAC*	+
	*srfAB*	+
Fengycin	*fenB*	+
	*fenC*	+

+ and − represent the presence or absence of each gene, respectively.

**Table 2:** Antifungal activity of thirty fractions against *A. alternata* using an Oxford cup.

Fractions	Inhibition	Fractions	Inhibition	Fractions	Inhibition
1 min	−	11 min	−	21 min	−
2 min	−	12 min	−	22 min	+
3 min	−	13 min	−	23 min	+
4 min	−	14 min	+	24 min	+
5 min	−	15 min	+	25 min	+
6 min	−	16 min	+	26 min	+
7 min	−	17 min	+	27 min	+
8 min	−	18 min	+	28 min	+
9 min	−	19 min	+	29 min	+
10 min	−	20 min	+	30 min	−

+ and − represent the presence or absence of inhibition, respectively.

**Table 3:** Composition of the control and experimental groups.

Treatment	Liquid Composition
CK	6 mL Spore suspension of *A. alternata* (10^5^ CFU/mL) + 12 mL PDB medium
Bacterial suspension of XJ-BV2007	6 mL Spore suspension of *A. alternata* (10^5^ CFU/mL) + 6 mL XJ-BV2007 bacterial suspension (10^5^ CFU/mL) + 6 mL PDB medium
Bacteria-free filtrate of XJ-BV2007	6 mL Spore suspension of *A. alternata* (10^5^ CFU/mL) + 6 mL XJ-BV2007 bacteria-free filtrate + 6 mL PDB medium

**Table 4:** Primers used for amplification of the lipopeptide genes.

No.	Gene		Primer Sequence (5′-3′)	Length (bp)	Lipopeptide
1	*SrfA* [44]	F’	AGAGCACATTGAGCGTTACAAA	626	Surfaction
		R’	CAGCATCTCGTTCAACTTTCAC		
2	*ItuD* [43]	F’	ATG AAC AAT CTT GCC TTT TTA	1203	Iturin
		R’	TTA TTT TAA AAT CCG CAA TT		
3	*ItuA* [43]	F’	TGCCAGACAGTATGAGGCAG	885	Iturin
		R’	CATGCCGTATCCACTGTGAC		
4	*ItuC* [46]	F’	CCCCCTCGGTCAAGTGAATA	594	Iturin
		R’	TTGGTTAAGCCCTGATGCTC		
5	*FenB* [46]	F’	CCTGGAGAAAGAATATACCGTACCY	670	Fengycin
		R’	GCTGGTTCAGTTKGATCACAT		
6	*BamC* [46]	R’	AGTAAATGAACGCGCCAATC	957	Bacillomycin D
		F’	CCCTCTCCTGCCACATAGAG		
7	*SrfAB* [44]	F’	GTTCTCGCAGTCCAGCAGAAG	308	Surfaction
		R’	GCCGAGCGTATCCGTACCGAG		
8	*BamD* [46]	F’	GCTGGTTCAGTTKGATCACAT	482	Bacillomycin D
		R’	TTGAAYGTCAGYGCSCCTTT		
9	*FenC* [46]	F’	CCCATCCGACYGTAGAAG	820	Fengycin
		R’	GTGTAAGCRGCAAGYAGCAC		

## Data Availability

Not applicable.

## Supplementary Materials

The following supporting information can be downloaded at: https://www.mdpi.com/article/10.3390/toxins15010053/s1, Table S1: Lipopeptides detected by UPLC IMS QTOF MS. Figure S1: The (A) CK (B) XJ-BV2007 UPLC-MSMS-chromatograms of TeA in tomato. Figure S2: The electropherogram of PCR detection with lipopeptides biosynthesis genes. Figure S3: The UPLC IMS QTOF MS chromatogram for detected lipopeptides at 14 min. Figure S4: The UPLC IMS QTOF MS chromatogram for detected lipopeptides at 15 min. Figure S5: The UPLC IMS QTOF MS chromatogram for detected lipopeptides at 16 min. Figure S6: The UPLC IMS QTOF MS chromatogram for detected lipopeptides at 17 min. Figure S7: The UPLC IMS QTOF MS chromatogram for detected lipopeptides at 18 min. Figure S8: The UPLC IMS QTOF MS chromatogram for detected lipopeptides at 19 min. Figure S9: The UPLC IMS QTOF MS chromatogram for detected lipopeptides at 20 min. Figure S10: The UPLC IMS QTOF MS chromatogram for detected lipopeptides at 22 min. Figure S11: The UPLC IMS QTOF MS chromatogram for detected lipopeptides at 23 min. Figure S12: The UPLC IMS QTOF MS chromatogram for detected lipopeptides at 24 min. Figure S13: The UPLC IMS QTOF MS chromatogram for detected lipopeptides at 25 min. Figure S14: The UPLC IMS QTOF MS chromatogram for detected lipopeptides at 26 min. Figure S15: The UPLC IMS QTOF MS chromatogram for detected lipopeptides at 27 min. Figure S16: The UPLC IMS QTOF MS chromatogram for detected lipopeptides at 28 min. Figure S17: The UPLC IMS QTOF MS chromatogram for detected lipopeptides at 29 min.
