# Supplementary material for: Effects of Bacillus amyloliquefaciens XJ-BV2007 on Growth of Alternaria alternata and Production of Tenuazonic Acid
Source: Toxins (Basel). 2023 Jan 7;15(1):53. doi: 10.3390/toxins15010053 (PMC9867350; doi:10.3390/toxins15010053)
Supplement: Supplementary file 1 [file toxins-15-00053-s001.zip › toxins-2130027-supplementary.pdf]

Supplementary Materials

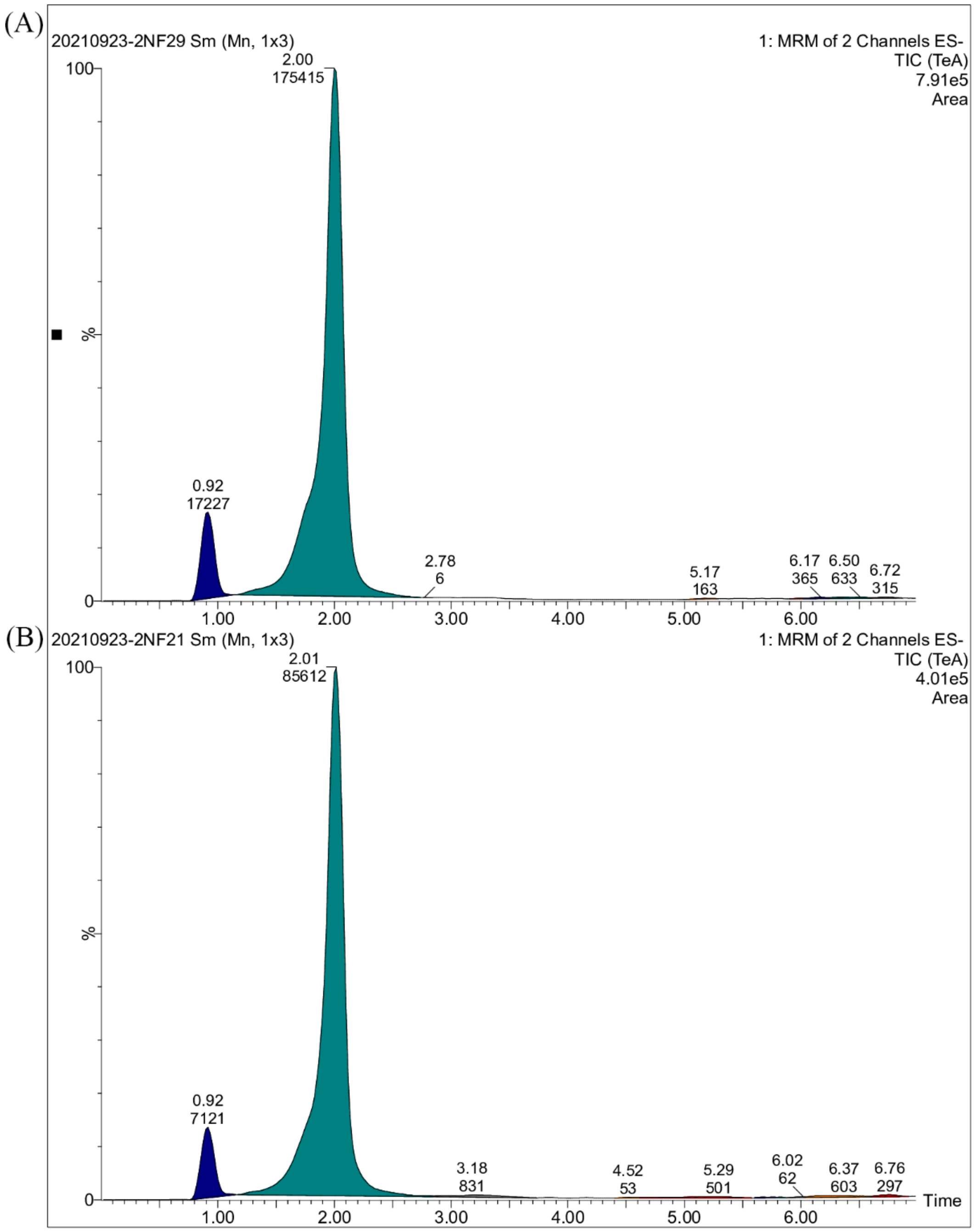

Figure S1. The (A) CK (B) XJ-BV2007 UPLC-MSMS-chromatograms of TeA in tomato.

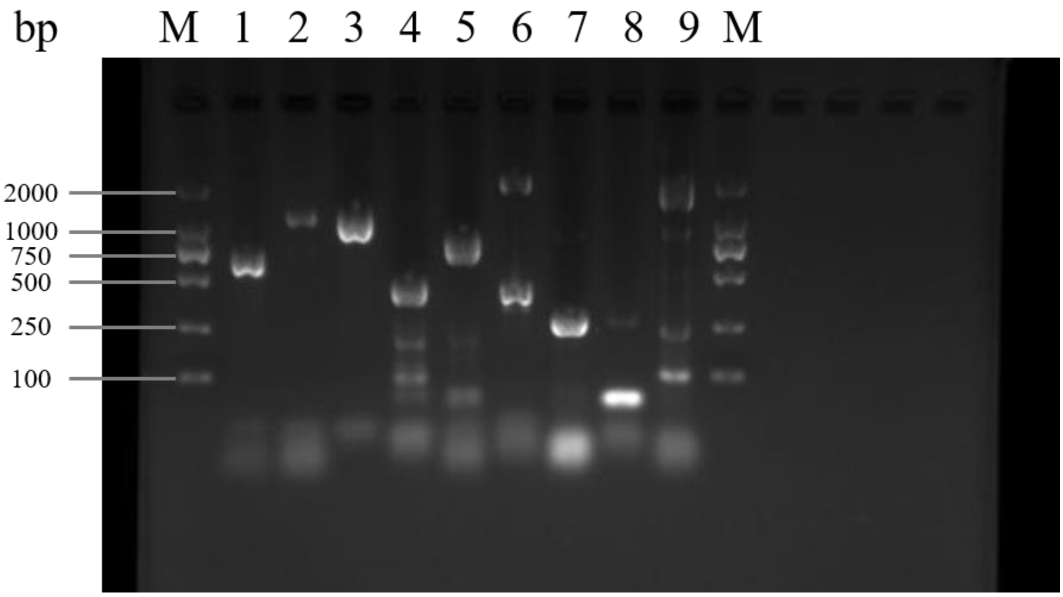

Figure S2. The electrophorogram of PCR detection with lipopeptides biosynthesis genes. Lane M 2000 bp DNA Ladder; Lane 1, Surfaction gene SrfA; Lane 2, iturin gene ituD; Lane 3, Iturin gene ItuA; Lane 4, Iturin gene ItuC; Lane 5, Fengycin gene FenB; Lane 6, Bacillomycin D gene BamC; Lane 7, Surfaction gene SrfAB; Lane 8, Bacillomycin D gene BamD; Lane 9, Fengycin gene FenC.

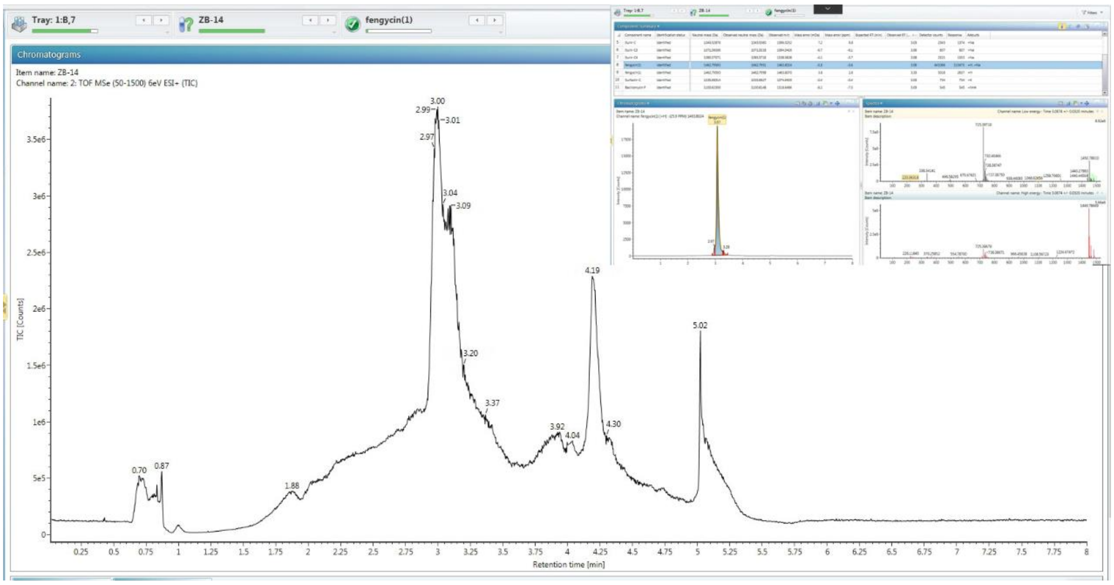

Figure S3. The UPLC IMS QTOF MS chromatogram for detected lipopeptides at 14 min

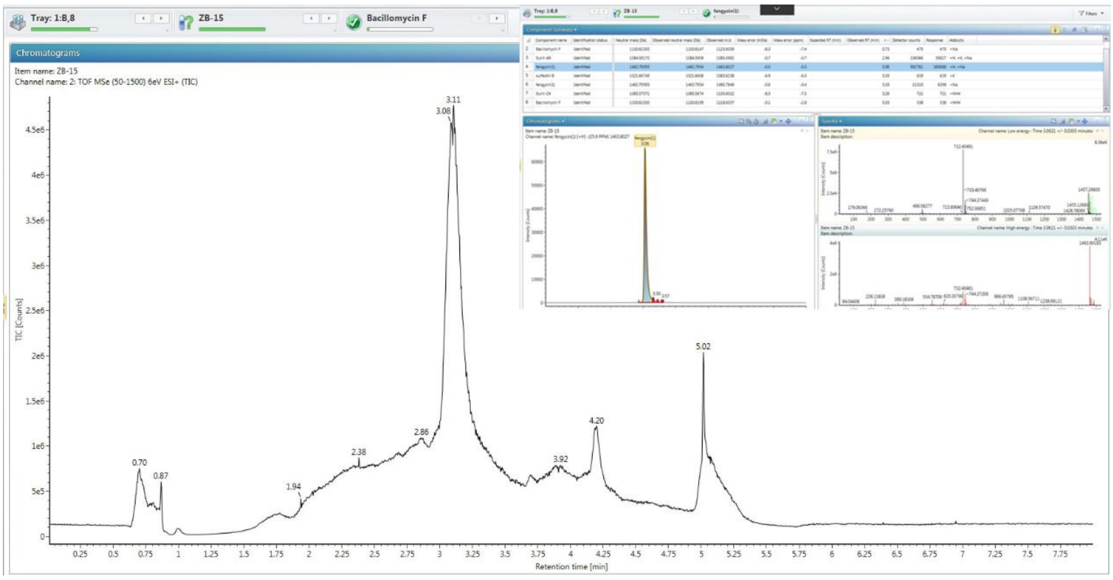

Figure S4. The UPLC IMS QTOF MS chromatogram for detected lipopeptides at 15 min

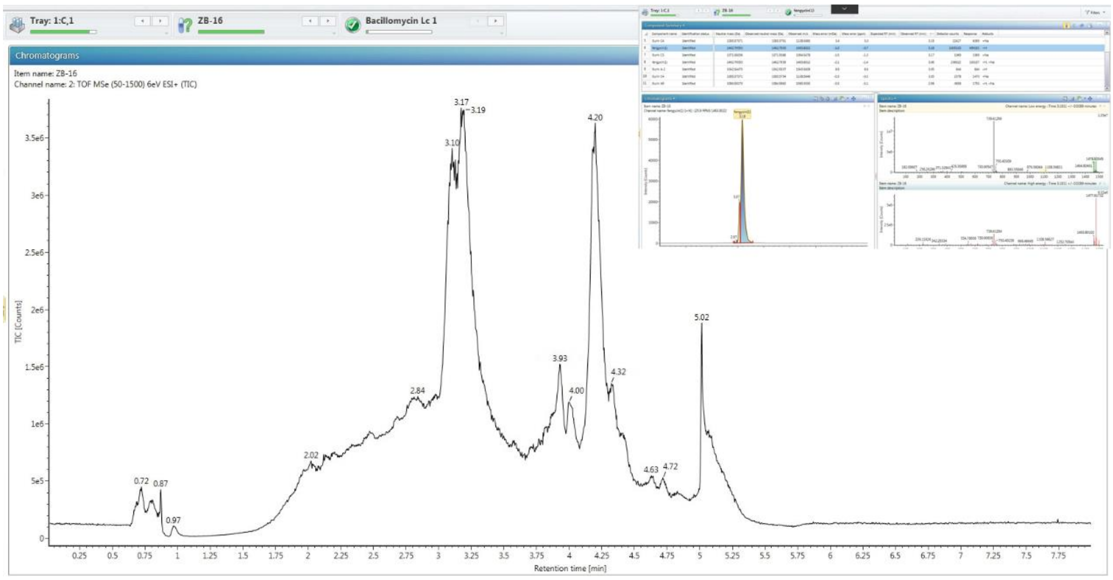

Figure S5. The UPLC IMS QTOF MS chromatogram for detected lipopeptides at 16 min

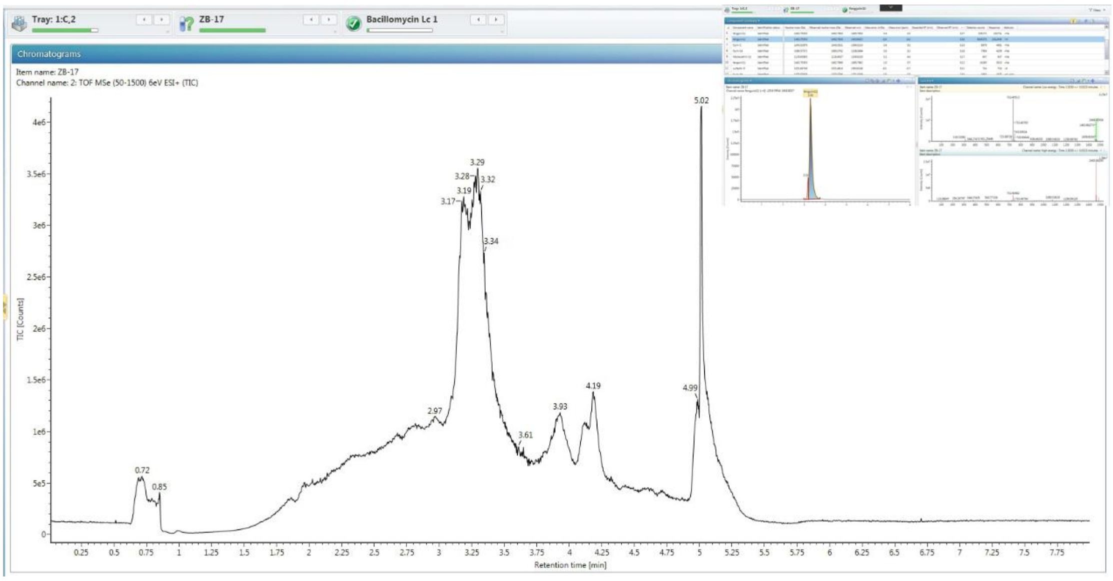

Figure S6. The UPLC IMS QTOF MS chromatogram for detected lipopeptides at 17 min

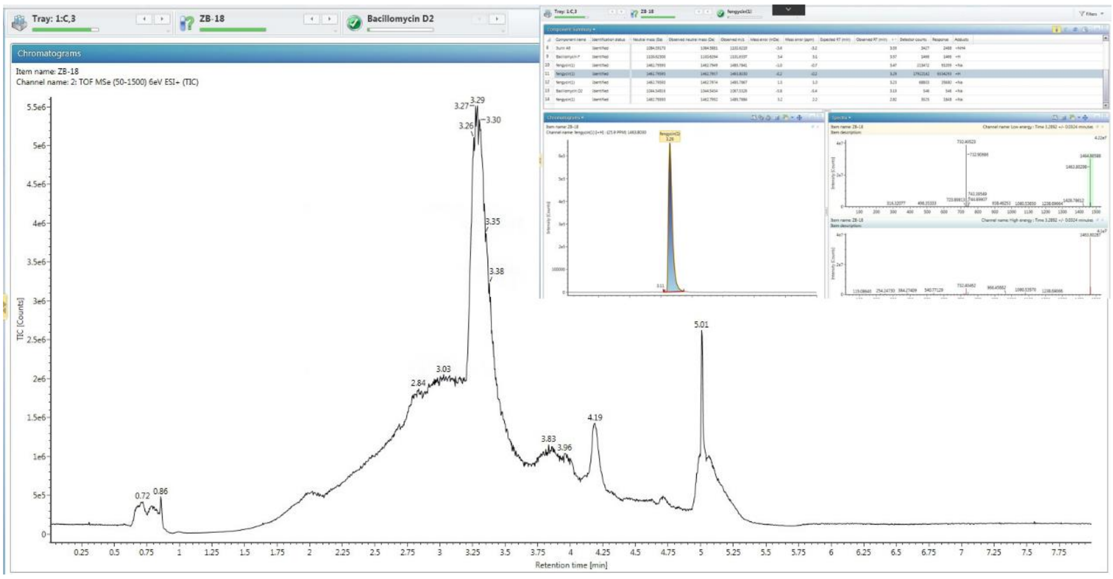

Figure S7. The UPLC IMS QTOF MS chromatogram for detected lipopeptides at 18 min

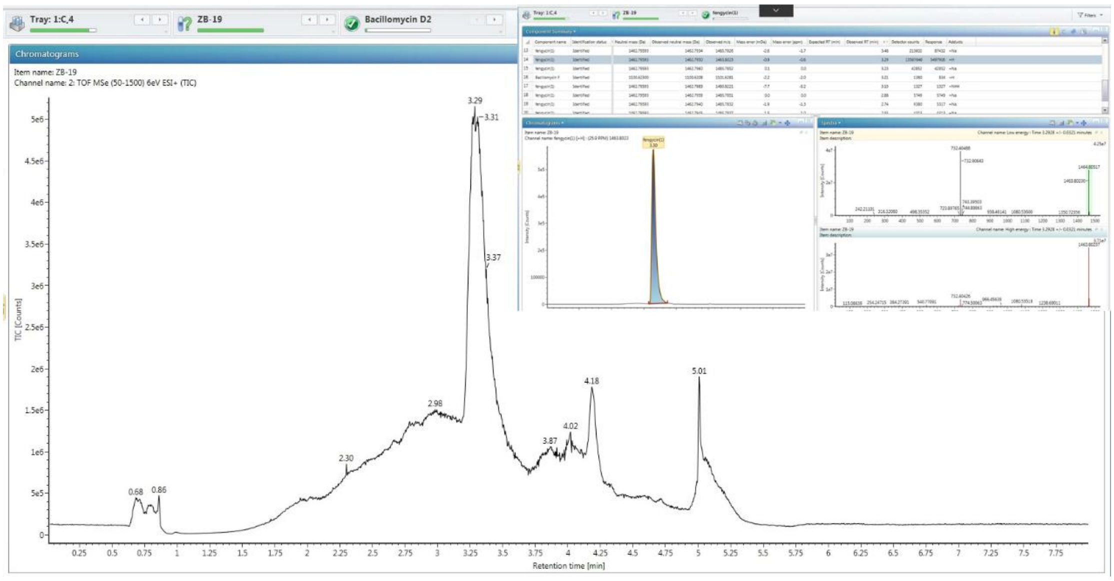

Figure S8. The UPLC IMS QTOF MS chromatogram for detected lipopeptides at 19 min

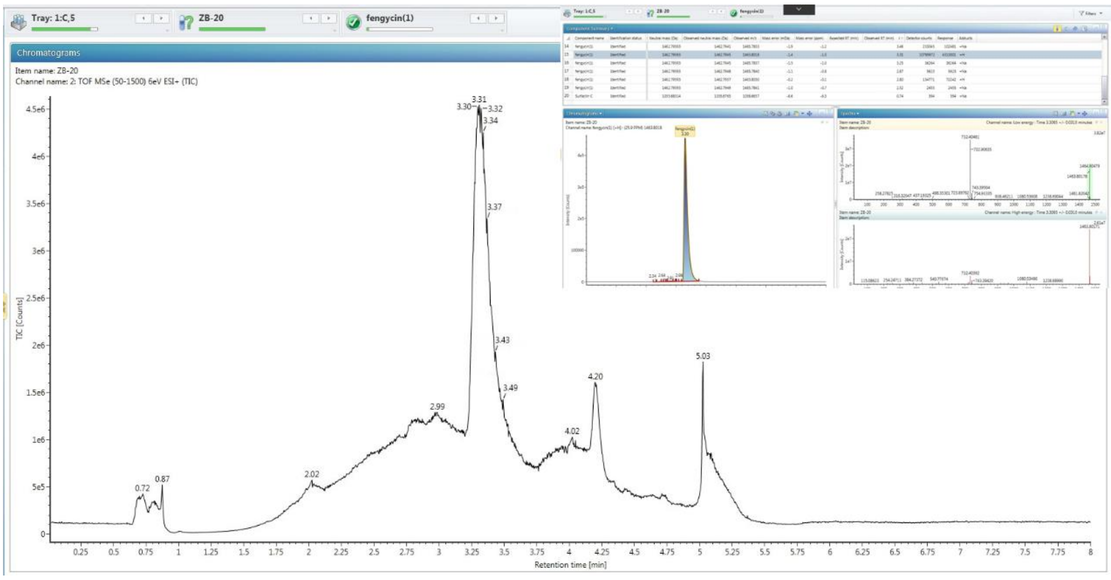

Figure S9. The UPLC IMS QTOF MS chromatogram for detected lipopeptides at 20 min

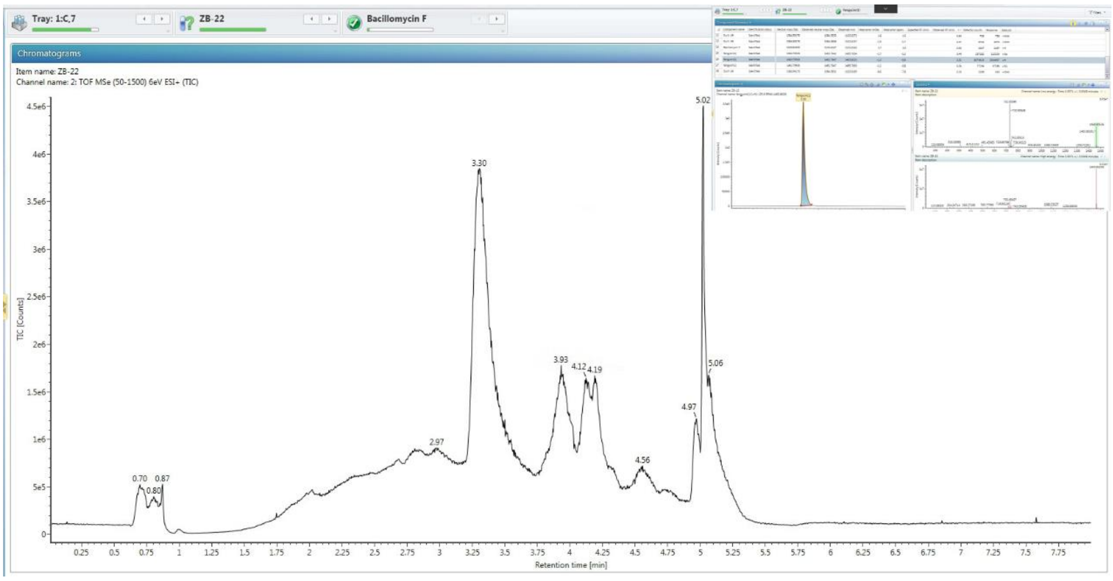

Figure S10. The UPLC IMS QTOF MS chromatogram for detected lipopeptides at 22 min

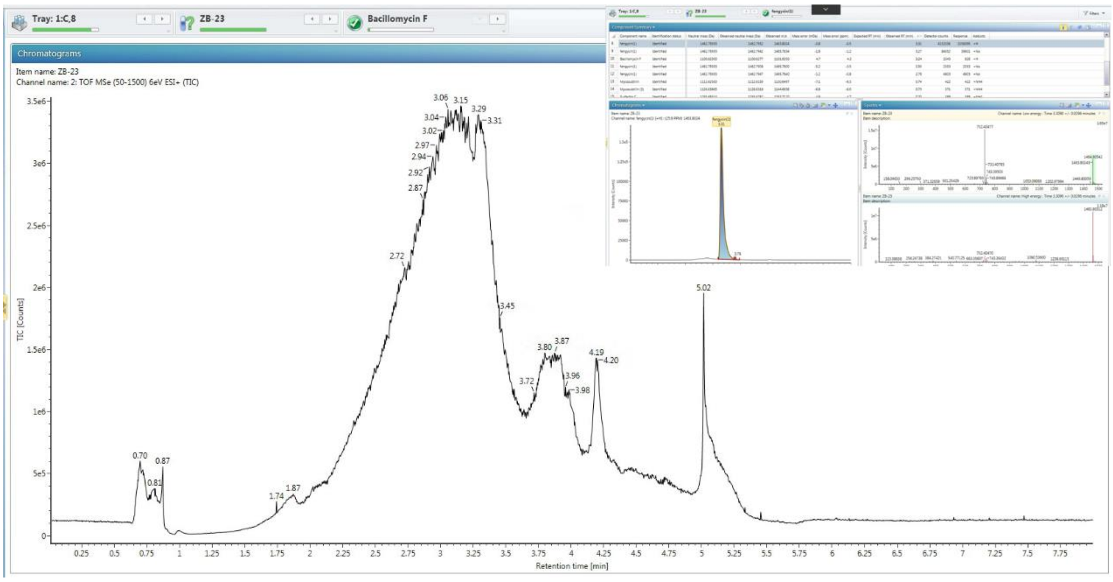

Figure S11. The UPLC IMS QTOF MS chromatogram for detected lipopeptides at 23 min

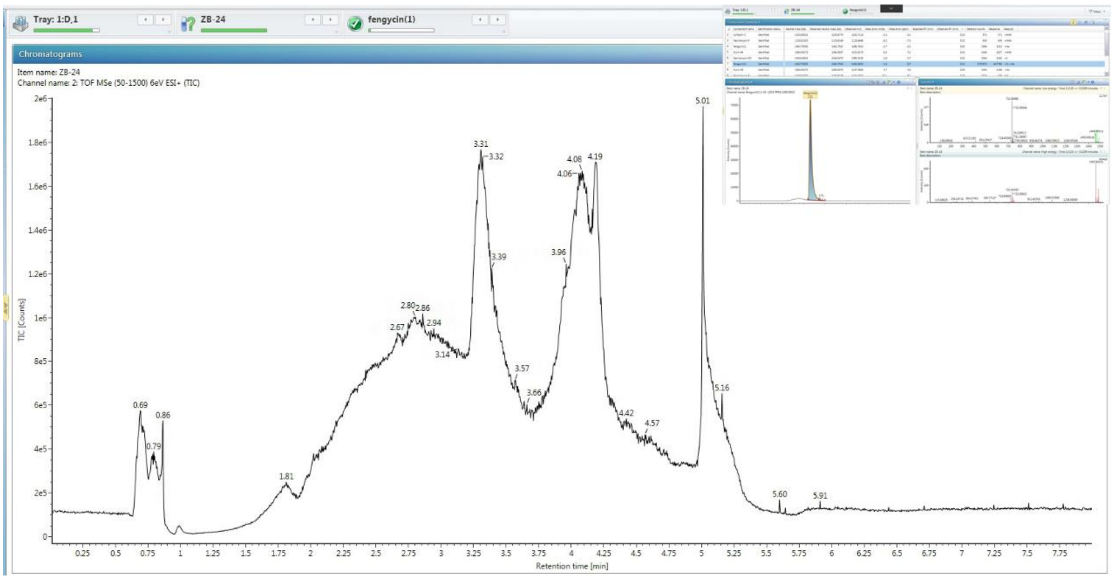

Figure S12. The UPLC IMS QTOF MS chromatogram for detected lipopeptides at 24 min

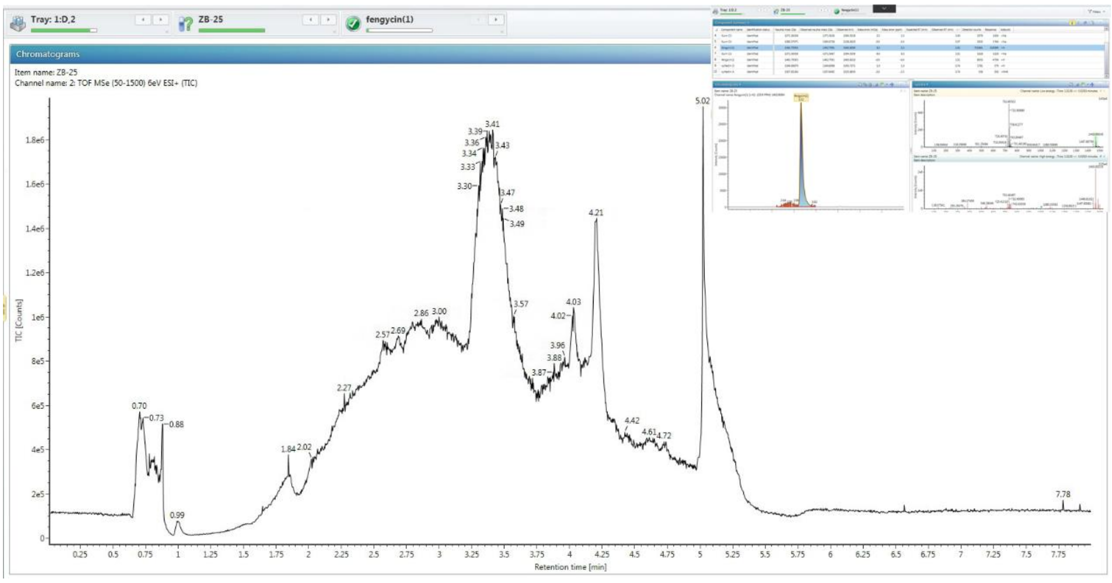

Figure S13. The UPLC IMS QTOF MS chromatogram for detected lipopeptides at 25 min

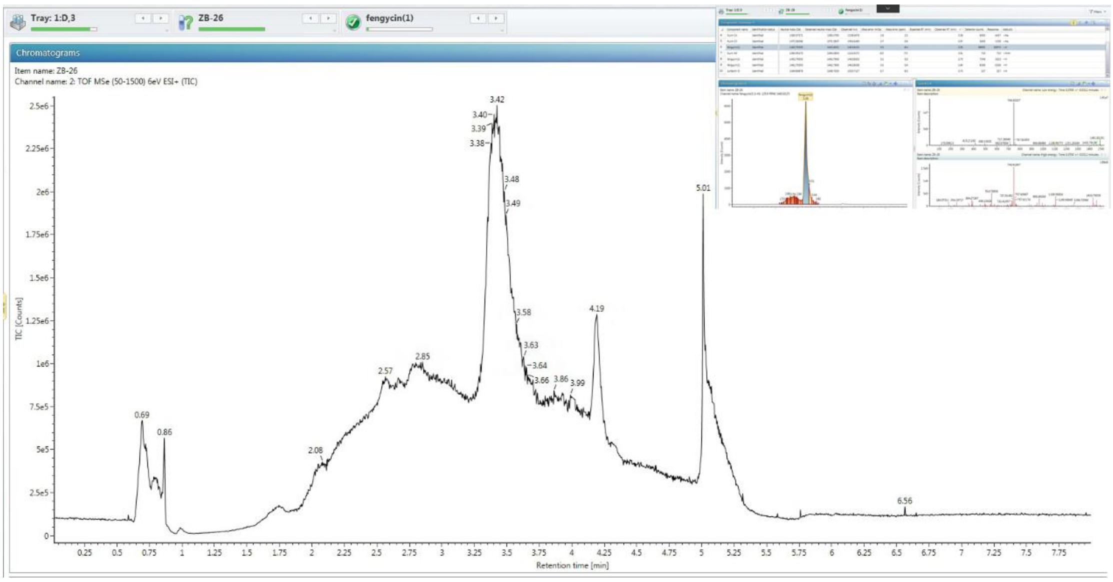

Figure S14. The UPLC IMS QTOF MS chromatogram for detected lipopeptides at 26 min

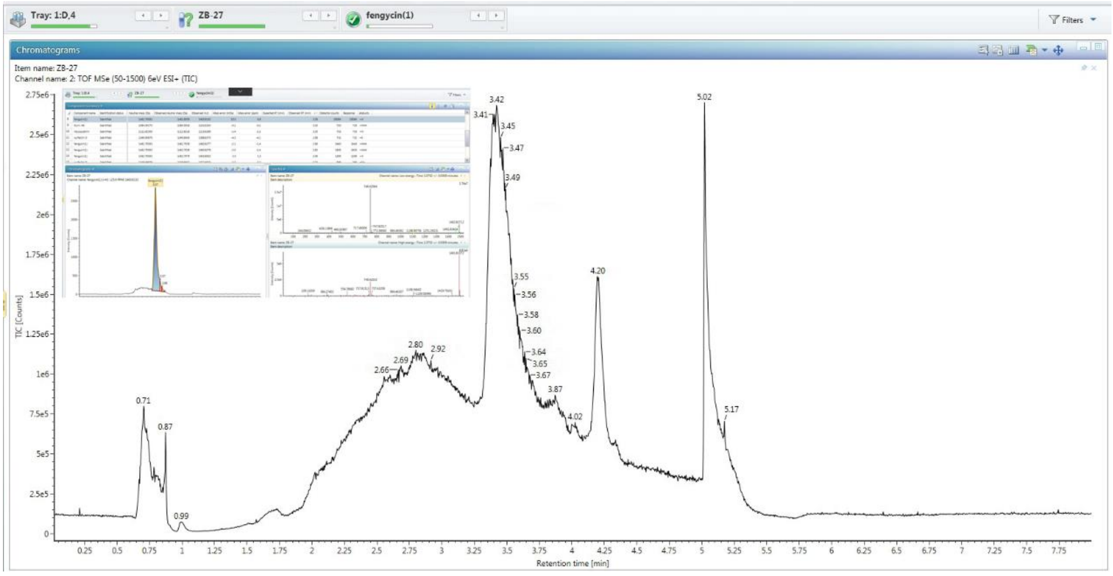

Figure S15. The UPLC IMS QTOF MS chromatogram for detected lipopeptides at 27 min

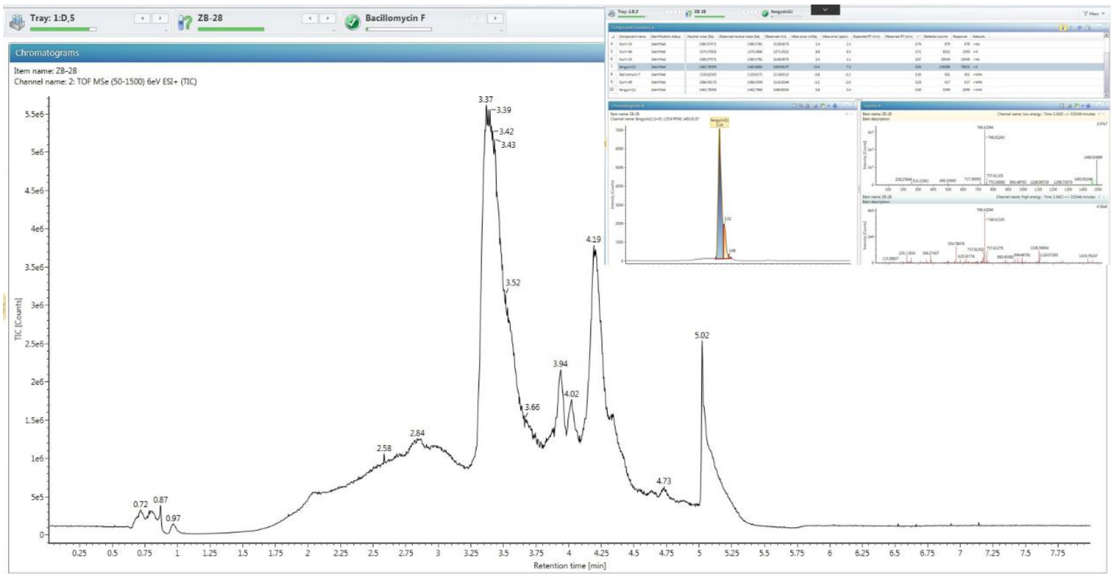

Figure S16. The UPLC IMS QTOF MS chromatogram for detected lipopeptides at 28 min

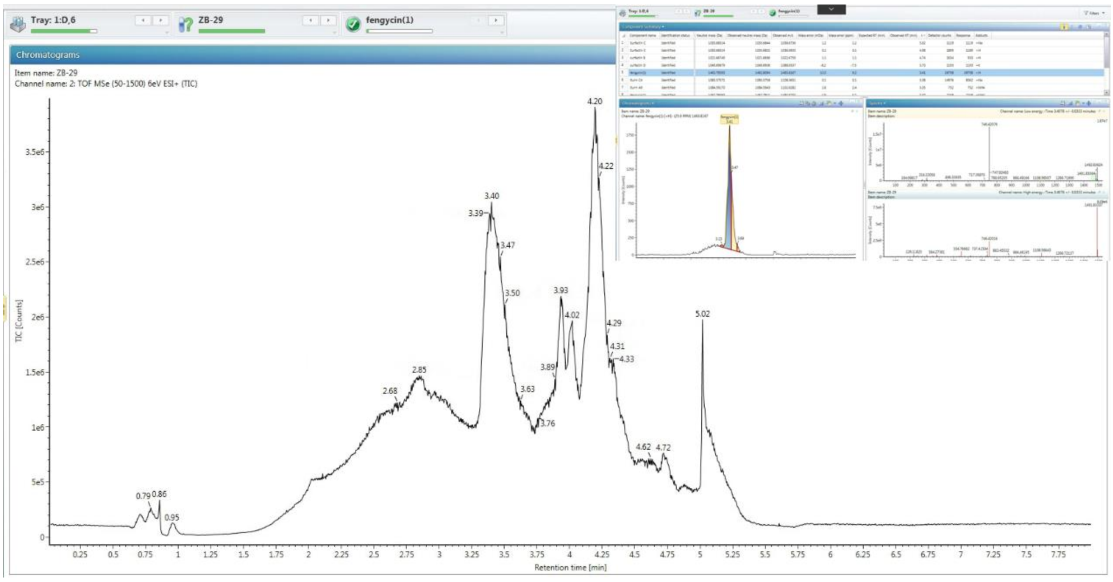

Figure S17. The UPLC IMS QTOF MS chromatogram for detected lipopeptides at 29 min

Table S1 Lipopeptides detected by UPLC IMS QTOF MS

Fractions	Component name	Molecular formula	Identification status	Expected mass (Da)	Observed mass (Da)	Observed m/z	Mass error (mDa)	Mass error (ppm)	Observed RT (min)	Detector counts	Response	Adducts
1 min	Iturin A4	$C_{49}H_{76}N_{12}O_{14}$	Identified	1056.5604	1056.5507	1095.5139	-9.672	-8.829	2.16	2825.18	1549.54	+K
2 min	Iturin A4	$C_{49}H_{76}N_{12}O_{14}$	Identified	1056.5604	1056.5614	1057.5687	1.042	0.985	2.67	7455.32	2202.45	+H
3 min	Surfactin A	$C_{51}H_{89}N_{7}O_{13}$	Identified	1007.6518	1007.6516	1008.6589	-0.196	-0.194	4.60	17987.72	7569.68	+H
4 min	Iturin A4	$C_{49}H_{76}N_{12}O_{14}$	Identified	1056.5604	1056.5540	1095.5172	-6.347	-5.794	1.87	4701.91	4701.91	+K
5 min	Iturin A2	$C_{48}H_{74}N_{12}O_{14}$	Identified	1042.5447	1042.5530	1081.5161	8.212	7.594	1.89	54644.86	28702.78	+K
6 min	Iturin A2	$C_{48}H_{74}N_{12}O_{14}$	Identified	1042.5447	1042.5478	1081.5110	3.099	2.866	1.90	7978.92	4626.82	+K
7 min	Fengycin	$C_{72}H_{110}N_{12}O_{20}$	Identified	1462.7959	1462.7940	1463.8013	-1.893	-1.293	3.32	6963.31	3002.95	+H
8 min	Iturin A2	$C_{48}H_{74}N_{12}O_{14}$	Identified	1042.5447	1042.5435	1043.5508	-1.221	-1.171	2.56	1617743.85	587235.94	+H
9 min	Iturin A4	$C_{49}H_{76}N_{12}O_{14}$	Identified	1056.5604	1056.5601	1057.5674	-0.247	-0.234	2.67	1991735.99	707317.63	+H
10 min	Iturin A4	$C_{49}H_{76}N_{12}O_{14}$	Identified	1056.5604	1056.5603	1057.5676	-0.061	-0.057	2.67	1880796.57	620264.06	+H
11 min	Iturin A6	$C_{50}H_{78}N_{12}O_{14}$	Identified	1070.5760	1070.5753	1071.5825	-0.794	-0.741	2.81	38629.37	10761.06	+H

12 min	Iturin A6	$C_{50}H_{78}N_{12}O_{14}$	Identified	1070.5760	1070.5756	1071.5829	-0.448	-0.418	2.84	1127653.35	355980.91	+H
13 min	Iturin A6	$C_{50}H_{78}N_{12}O_{14}$	Identified	1070.5760	1070.5757	1071.5829	-0.372	-0.347	2.85	542399.64	162019.52	+H
14 min	Fengycin	$C_{72}H_{110}N_{12}O_{20}$	Identified	1462.7959	1462.7951	1463.8024	-0.832	-0.568	3.09	441066.31	110475.26	+H
15 min	Fengycin	$C_{72}H_{110}N_{12}O_{20}$	Identified	1462.7959	1462.7954	1463.8027	-0.508	-0.347	3.06	992780.52	383687.88	+H
16 min	Fengycin	$C_{72}H_{110}N_{12}O_{20}$	Identified	1462.7959	1462.7950	1463.8022	-0.977	-0.668	3.18	1405319.75	499191.69	+H
17 min	Fengycin	$C_{72}H_{110}N_{12}O_{20}$	Identified	1462.7959	1462.7954	1463.8027	-0.484	-0.331	3.30	5604378.00	2012406.00	+H
18 min	Fengycin	$C_{72}H_{110}N_{12}O_{20}$	Identified	1462.7959	1462.7957	1463.8030	-0.228	-0.156	3.29	17922162.00	6534292.50	+H
19 min	Fengycin	$C_{72}H_{110}N_{12}O_{20}$	Identified	1462.7959	1462.7950	1463.8023	-0.907	-0.619	3.29	15567646.00	5497906.00	+H
20 min	Fengycin	$C_{72}H_{110}N_{12}O_{20}$	Identified	1462.7959	1462.7945	1463.8018	-1.434	-0.979	3.31	10789972.00	4310001.00	+H
21 min	Surfactin A	$C_{51}H_{89}N_{7}O_{13}$	Identified	1007.6518	1007.6511	1008.6584	-0.723	-0.717	4.55	380013.12	176134.27	+H
22 min	Fengycin	$C_{72}H_{110}N_{12}O_{20}$	Identified	1462.7959	1462.7947	1463.8020	-1.204	-0.822	3.31	8073618.50	2944697.25	+H
23 min	Fengycin	$C_{72}H_{110}N_{12}O_{21}$	Identified	1462.7959	1462.7952	1463.8024	-0.780	-0.533	3.31	4152036.00	1556098.50	+H
24 min	Fengycin	$C_{72}H_{110}N_{12}O_{22}$	Identified	1462.7959	1462.7949	1463.8022	-1.048	-0.716	3.31	1752552.87	647779.88	+H

25 min	Fengycin	$C_{72}H_{110}N_{12}O_{23}$	Identified	1462.7959	1462.7991	1463.8064	3.150	2.152	3.32	701860.56	319088.78	+H
26 min	Fengycin	$C_{72}H_{110}N_{12}O_{24}$	Identified	1462.7959	1462.8052	1463.8125	9.315	6.364	3.35	88991.52	56969.60	+H
27 min	Fengycin	$C_{72}H_{110}N_{12}O_{25}$	Identified	1462.7959	1462.8059	1463.8132	10.011	6.839	3.38	28084.07	28084.07	+H
28 min	Fengycin	$C_{72}H_{110}N_{12}O_{26}$	Identified	1462.7959	1462.8064	1463.8137	10.445	7.135	3.34	103586.43	70914.91	+H
29 min	Fengycin	$C_{72}H_{110}N_{12}O_{27}$	Identified	1462.7959	1462.8094	1463.8167	13.465	9.199	3.41	26738.10	26738.10	+H
30 min	Fengycin	$C_{72}H_{110}N_{12}O_{28}$	Identified	1462.7959	1462.7951	1480.8289	-0.834	-0.563	2.84	2139.91	2139.91	+NH4
